# Engineering *Escherichia coli* for high-yield geraniol production with biotransformation of geranyl acetate to geraniol under fed-batch culture

**DOI:** 10.1186/s13068-016-0466-5

**Published:** 2016-03-11

**Authors:** Wei Liu, Xin Xu, Rubing Zhang, Tao Cheng, Yujin Cao, Xiaoxiao Li, Jiantao Guo, Huizhou Liu, Mo Xian

**Affiliations:** CAS Key Laboratory of Bio-Based Materials, Qingdao Institute of Bioenergy and Bioprocess Technology, Chinese Academy of Sciences, Qingdao, 266101 China; Key Laboratory of Green Process and Engineering, Institute of Process Engineering, Chinese Academy of Sciences, Beijing, 10090 China; University of Chinese Academy of Sciences, Beijing, 100049 China; Department of Chemistry, University of Nebraska–Lincoln, Lincoln, NE 68588 USA

**Keywords:** Geraniol, Geranyl acetate, Fed-batch fermentation, Acetylesterase, Engineered *Escherichia coli*

## Abstract

**Background:**

Geraniol is an acyclic monoterpene alcohol, which exhibits good prospect as a gasoline alternative. Geraniol is naturally encountered in plants at low concentrations and an attractive target for microbial engineering. Geraniol has been heterologously produced in *Escherichia coli*, but the low titer hinders its industrial applications. Moreover, bioconversion of geraniol by *E. coli* remains largely unknown.

**Results:**

Recombinant overexpression of *Ocimum basilicum* geraniol synthase, *Abies grandis* geranyl diphosphate synthase, and a heterotic mevalonate pathway in *E. coli* BL21 (DE3) enabled the production of up to 68.6 ± 3 mg/L geraniol in shake flasks. Initial fed-batch fermentation only increased geraniol production to 78.8 mg/L. To further improve the production yield, the fermentation conditions were optimized. Firstly, 81.4 % of volatile geraniol was lost during the first 5 h of fermentation in a solvent-free system. Hence, isopropyl myristate was added to the culture medium to form an aqueous-organic two-phase culture system, which effectively prevented volatilization of geraniol. Secondly, most of geraniol was eventually biotransformed into geranyl acetate by *E. coli*, thus decreasing geraniol production. For the first time, we revealed the role of acetylesterase (Aes, EC 3.1.1.6) from *E. coli* in hydrolyzing geranyl acetate to geraniol, and production of geraniol was successfully increased to 2.0 g/L under controlled fermentation conditions.

**Conclusions:**

An efficient geraniol production platform was established by overexpressing several key pathway proteins in engineered *E. coli* strain combined with a controlled fermentation system. About 2.0 g/L geraniol was obtained using our controllable aqueous-organic two-phase fermentation system, which is the highest yield to date. In addition, the interconversion between geraniol and geranyl acetate by *E. coli* was first elucidated. This study provided a new and promising strategy for geraniol biosynthesis, which laid a basis for large-scale industrial application.

## Background

Monoterpene geraniol (*trans*-isomer of 3,7-dimethyl-2, 6-octadiene-1-ol), which is emitted from flowers, has been widely applied in perfume, pharmaceutical, and other industries [1–3]. As a gasoline alternative, geraniol is superior to ethanol due to low hygroscopicity, high energy content, and relatively low volatility [4, 5]. Geraniol is derived from geranyl diphosphate (GPP) which is synthesized from either the mevalonate (MVA) pathway or the methylerythritol phosphate pathway in plants [6, 7]. However, geraniol has low economic value because it can only be extracted naturally from plants at very low concentrations. Large amounts of value-added products can be generated through the metabolic engineering of microbial hosts [8–10]. Unlike plants, microorganisms usually do not carry a specific GPP synthase (GPPS) and cannot make monoterpenes with the exception of a few winemaking *Saccharomyces cerevisiae* strains which manage to do so (5 mg/L monoterpenes) [11–13]. In recent years, geraniol has been successfully heterologously produced in *Escherichia coli* and *S. cerevisiae*. Mutations in farnesyl diphosphate synthase (FPPS) allow GPP release for monoterpene biosynthesis in recombinant microorganisms harboring monoterpene synthases [11, 14, 15]. A recent study demonstrated that GPP accumulation in yeast bearing mutated FPPS enabled geraniol formation in the absence of a heterologous geraniol synthase probably through endogenous dephosphorylation [11, 14]. Geraniol can also be generated even in the absence of specific GPPS or mutated FPPS in *E. coli* by simply overexpressing an *Ocimum basilicum* geraniol synthase (GES), although the GPP release mechanism remains unclear [16]. By co-overexpression of a FPPS mutant and GES in *S. cerevisiae*, 5 mg/L geraniol was obtained after 7 days of culture [11]. Production of geraniol was further increased to 36.04 mg/L in *S. cerevisiae* harboring both regulator gene *MAF1* and *GES* after 48 h of culture by overexpressing key rate-limiting enzymes of the MVA pathway [17]. So far, maximum geraniol (182.5 mg/L) has been produced by geraniol dehydrogenase mutant *E. coli* with the whole MVA pathway and GES after 48 h of culture [5]. However, the titer is still too low for industrial applications.

In addition, geraniol usually undergoes biotransformation to other terpenoids in aromatic plants, which influences the quality of distilled essential oils [18, 19]. The conversion of geraniol to trans-citral in *Cymbopogon flexuosus* leaves is catalyzed by NADP^+^-dependent geraniol dehydrogenase [20]. Similarly, some wine yeasts can modify the free terpenoid contents, although they only have limited capability to produce monoterpenoids [21–23]. *S. cerevisiae* is able to convert geraniol into citronellol under the catalysis of enzyme OYE2 [24], and ATF1 alcohol acetyltransferase is involved in the acetylation of geraniol during *S. cerevisiae* fermentation [24]. Unlike the extensive studies on yeast, the bioconversion of geraniol in *E. coli* has seldom been referred and only until recently, geraniol has been dehydrogenized and isomerized into other geranoids (nerol, neral, and geranial) in *E. coli* by enzyme YjgB [5].

Thereby motivated, we created an effective geraniol-biosynthesizing strain and developed a new high-performance fermentation strategy to increase geraniol production. In addition, we observed the interconversion between geraniol and geranyl acetate in *E. coli*. The mechanism by which geranyl acetate was hydrolyzed into geraniol was thus investigated and controlled to further increase geraniol production.

## Results and discussion

### Regulated biosynthesis of geraniol from glucose in *E. coli*

**Fig. 1:**
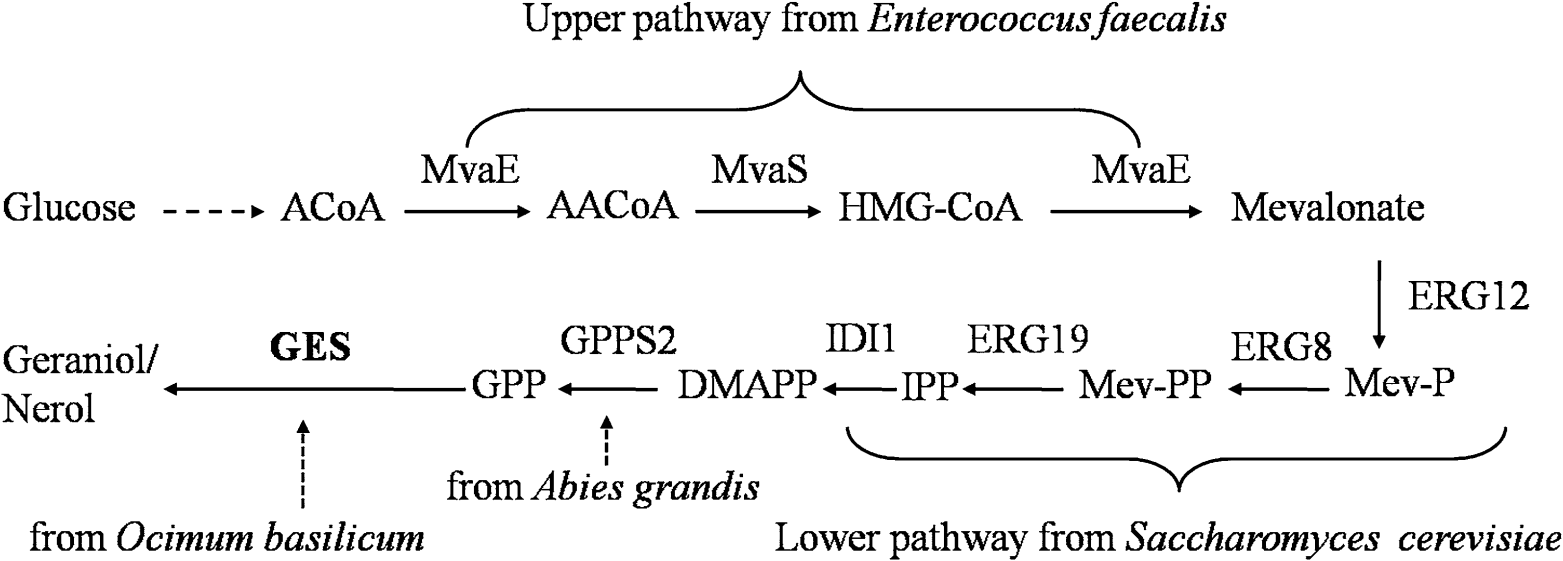
Production of geraniol via the MVA pathways used in this study. Enzymes involved in this pathway: MvaE, *E. faecalis* acetyl-CoA acetyltransferase/HMG-CoA reductase; MvaS, *E. faecalis* HMG-CoA synthase; ERG12, *S. cerevisiae* mevalonate kinase; ERG8, *S. cerevisiae* phosphomevalonate kinase; ERG19, *S. cerevisiae* mevalonate pyrophosphate decarboxylase; IDI1, *S. cerevisiae* IPP isomerase; GPPS2, *A. grandis* geranyl diphosphate synthase; GES, *O. basilicum* synthase was optimized to the preferred codon usage of *E. coli*. Pathway intermediates: A-CoA, acetyl-CoA; AA-CoA, acetoacetyl-CoA; HMG-CoA, hydroxymethylglutaryl-CoA; Mev-P, mevalonate 5-phosphate; Mev-PP, mevalonate pyrophosphate. *IPP* isopentenyl pyrophosphate, *DMAPP* dimethylallyl pyrophosphate, *GPP* geranyl diphosphate

A highly efficient strain LWG6 was constructed to produce geraniol from glucose in *E. coli*, comprising a heterotic MVA pathway from *Enterococcus faecalis* and *S. cerevisiae*, GPP synthase GPPS2 from *Abies grandis*, and codon-optimized GES from *O. basilicum*. This biosynthetic pathway (Fig. 1) was adapted from a previous study with genes from different origins [5]. It has previously been reported that GPP was efficiently synthesized by this heterotic MVA pathway and GPP synthase from *A. grandis* [25, 26]. After 48 h of shake-flask culture (OD_600_ = 2), 68.6 ± 3 mg/L geraniol was obtained from glucose by LWG6, while the control strain LWG10 without GES failed to produce geraniol. The geraniol production efficiency of strain LWG6 (34.3 mg/L/OD_600_) doubled that of strain GEOLW (about 16 mg/L/OD_600_) after 48 h of culture in flask [5], which followed a similar geraniol synthesis pathway to that of wild type *E. coli* MG 1655. Accordingly, LWG6 was a promising strain for geraniol synthesis.

### Geranyl acetate formation in *E. coli* under fed-batch fermentation condition

**Fig. 2:**
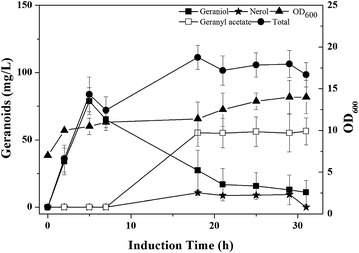
Fed-batch fermentation by LWG6 in a solvent-free system. Accumulation of total geranoids (*filled circle*) including geraniol (*filled square*), nerol (*circle*), and geranyl acetate (*square*). Induction was carried out when OD_600_ (*filled triangle*) reached about 10 using 0.5 mM IPTG. Results are the mean of three replicates with *error bars* representing the standard deviation

**Fig. 3:**
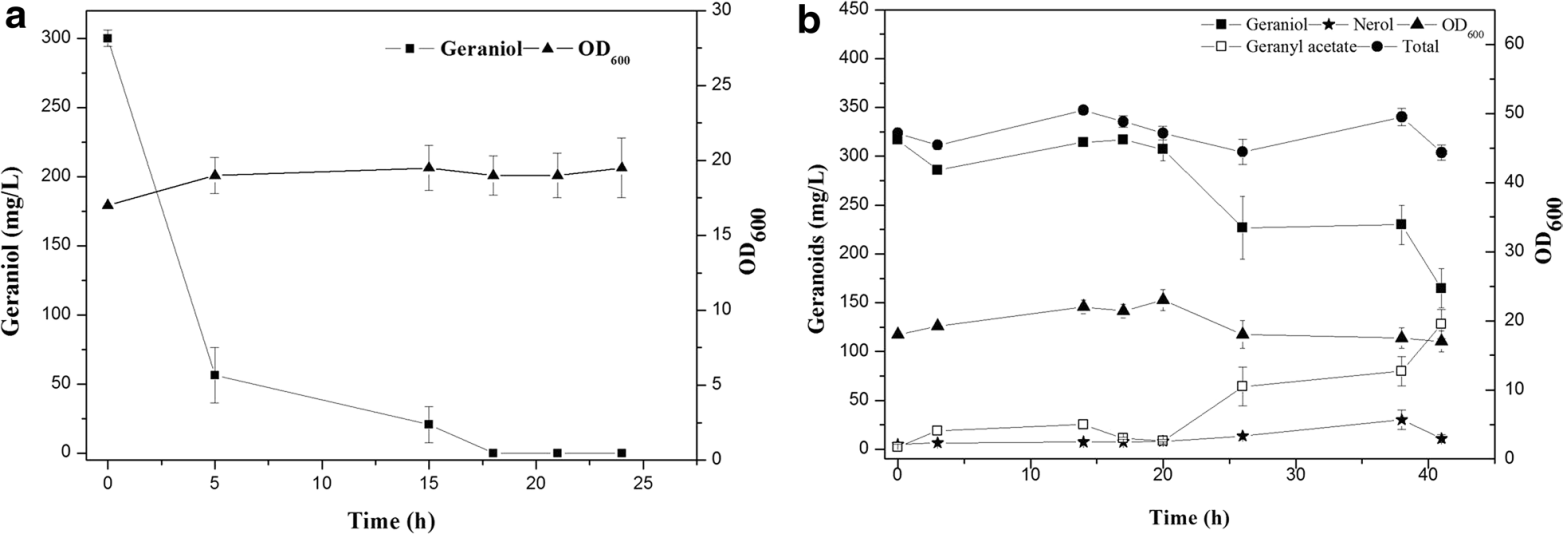
Geranyl acetate feeding experiments by *E. coli* BL21 (DE3). **a** Feeding experiments in a solvent-free system. **b** Feeding experiments in aqueous-organic two-phase culture system. Isopropyl myristate was added when OD_600_ (*filled triangle*) reached about 20 according to one over ten of the fermentation medium volume. Accumulation of total geranoids (*filled circle*) including geraniol (*filled square*), nerol (*circle*), and geranyl acetate (*square*). Results are the mean of three replicates with *error bars* representing the standard deviation

The fed-batch fermentation was carried out with LWG6 based on the results obtained above in shake flask. Geraniol accumulation was monitored over the course of fermentation (Fig. 2). The highest concentration of geraniol was just 78.8 mg/L after being induced by isopropyl *β*-d-thiogalactoside (IPTG) for 5 h. The low titer then plummeted to 12.9 mg/L after 24 h, which may be ascribed to the volatilization of geraniol during fermentation and the accumulated toxicity may further prevent its synthesis by engineered *E. coli* [27, 28]. To prove the volatility of geraniol during fermentation, an authentic geraniol standard was fed to the culture of *E. coli* BL21 (DE3) in a 5 L fermentor. As shown in Fig. 3a, 81.4 % of fed geraniol is lost during the first 5 h of fermentation, probably through volatilization. In order to prevent volatilization, isopropyl myristate that also can reduce monoterpene toxicity was added [27], forming an aqueous-organic two-phase culture system. With this system, the amount of fed geraniol was kept stable during 20 h of culture (Fig. 3b), which was conducive to geraniol fermentation.

Moreover, the dehydrogenation and isomerization of geraniol into other geranoids (nerol, neral, and geranial) may also be responsible for the loss of geraniol [5]. Only 10.8 mg/L nerol was detected at 18 h, which descended to zero at the end of fermentation (Fig. 2). Instead of nerol, neral, and geranial, a new compound appeared at 18 h of culture, which was identified as geranyl acetate by GC-MS analysis. At the end of fermentation, geranyl acetate accounted for 83.7 % of total geranoids.

The formation of geranyl acetate was analyzed. Firstly, *cat* gene in plasmid pSTV28 encoding chloramphenicol acetyltransferase (CAT) is known to be responsible for chloramphenicol resistance, which exhibits non-specific esterification activity toward esterification of geraniol into geranyl acetate [5]. Plasmid pACYDuet-1 used in our study also harbors CAT, forming geranyl acetate. Secondly, in a previous study, acetyltransferase ATF1 (EC 2.3.1.84) from *S. cerevisiae* mainly contributed to geranyl acetate synthesis [24]. It is highly possible that similar functional enzymes of o-acetyltransferase (EC 2.3.1.9) also exist in *E. coli* and cause the esterification of geraniol therein [29]. This postulation was supported by the fed experiment shown in Fig. 3b. Geraniol decreased after 20 h of culture along with the accumulation of geranyl acetate. After 40 h of culture, more than 40 % of fed geraniol was converted into geranyl acetate (128.3 mg/L) by *E. coli* BL21 (DE3). Nerol was also detected but the total production was lower than 8 %, suggesting that geraniol was not lost mainly through dehydrogenation in *E. coli* BL21 (DE3) under such fed-batch conditions. Geranial and neral were not found and the amounts of total geranoids (geraniol, nerol, and geranyl acetate) were relatively stable during fermentation.

### Conversion of geranyl acetate to geraniol by AES from *E. coli*

Simple gene knockout may not effectively prevent geranyl acetate synthesis, so geraniol production can feasibly be augmented by converting geranyl acetate to geraniol in engineered *E. coli*. Acetylesterase GAE (EC 3.1.1.6) from *Cymbopogon martinii* is involved in the transformation of geranyl acetate into geraniol [18]. Acetylesterase (Aes, EC 3.1.1.6) also exists in *E. coli*, although its effect on geranyl acetate is still not clear [30]. In our study, Aes was overexpressed in *E. coli* BL21 (DE3), the activity of which was indicated by incubating geranyl acetate under defined conditions and monitoring the amount of produced geraniol with GC-MS. Since about 75 % of geranyl acetate was converted into geraniol after 2 h of incubation, *E. coli* was capable of geranyl acetate hydrolyzation. No geraniol was produced in the control experiment using boiled and denatured enzyme.

### Geraniol production under fed-batch fermentation with biotransformation of geranyl acetate to geraniol

**Fig. 4:**
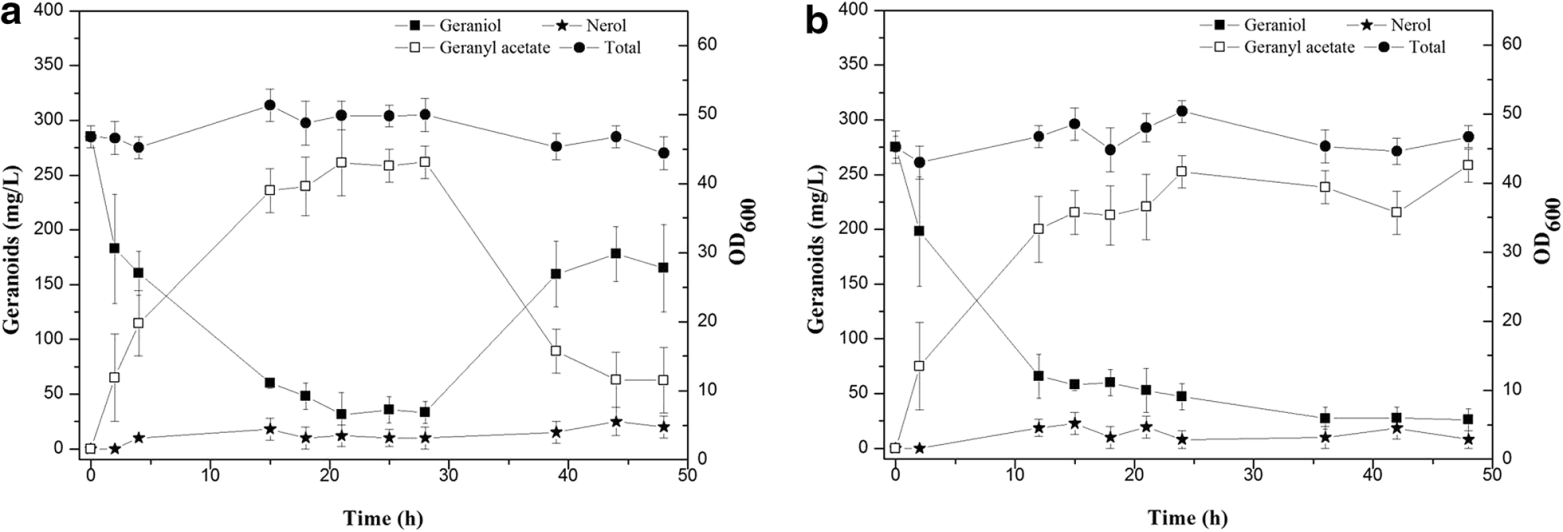
Geranyl acetate feeding experiments by LWG10. **a** Glucose addition was stopped after 28 h of culture. **b** The residual glucose was maintained below 5 g/L by a feeding solution containing 800 g/L glucose at appropriate rates. Isopropyl myristate was added when OD_600_ reached about 20 according to one over ten of the fermentation medium volume. Accumulation of total geranoids (*filled circle*) including geraniol (*filled square*), nerol (*circle*), and geranyl acetate (*square*). Results are the mean of three replicates with *error bars* representing the standard deviation

Considering the effect of Aes, geraniol production can feasibly be increased through biotransformation of geranyl acetate to geraniol during fermentation. It can be allowed by overexpression of Aes in LWG6 which, however, further burdens the cell metabolism system, since eight heterologous genes have already been designed to be overexpressed. Moreover, the above transformation can be realized by regulating fermentation. In the absence of glucose, *E. coli* cells reuse acetate [31], thereby facilitating the formation of geraniol catalyzed by Aes. Geraniol feeding experiment was used to identify whether geranyl acetate, which was esterified from geraniol, can reproduce geraniol by this glucose starvation strategy. The control strain LWG10 was used in feeding experiment to ensure similar conversion of geraniol to geranyl acetate. As shown in Fig. 4a, fed geraniol is lost quickly by LWG10 and about 86 % of geraniol is converted to geranyl acetate at 21 h. From 21 to 28 h, the geraniol amount remained stable, suggesting the reaction between geraniol and geranyl acetate reached equilibrium. Then glucose supply was stopped at 28 h, and geraniol production rose from 33 to 160 mg/L at 39 h when residual glucose was exhausted, probably because the reuse of acetate disturbed the reaction balance and moved the reaction toward geraniol formation under the catalysis of Aes. For comparison, glucose was continuously added and most of geraniol was quickly converted to geranyl acetate that was kept at a low concentration thereafter (Fig. 4b).

**Fig. 5:**
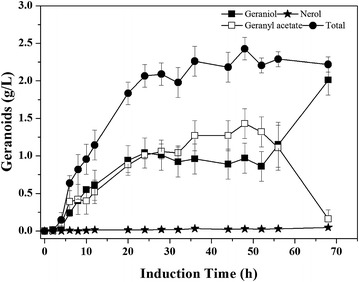
Geraniol production under fed-batch fermentation with biotransformation of geranyl acetate to geraniol. Accumulation of total geranoids (*filled circle*) including geraniol (*filled square*), nerol (*circle*), and geranyl acetate (*square*). Induction was carried out when OD_600_ reached about 20 using 0.5 mM IPTG. Isopropyl myristate was added 4 h after IPTG induction according to one over ten of the fermentation medium volume, and glucose addition was stopped after 48 h of culture. Results are the mean of three replicates with *error bars* representing the standard deviation

Thus, a new fermentation strategy was established based on the above results. First, isopropyl myristate was added to form an aqueous-organic two-phase culture system. Second, glucose starvation was employed to convert geranyl acetate into geraniol. Therefore, this new fermentation condition was used with LWG6 (Fig. 5). At the beginning, both geraniol and geranyl acetate increased quickly and OD_600_ value of the bacterial culture increased from 20 to 32 rapidly. The titers of geraniol and geranyl acetate reached 1.04 and 1.01 g/L at 24 h of culture, respectively. During the next 24 h, geranyl acetate increased to 1.43 g/L with decreasing geraniol, suggesting that the strain no longer synthesized geraniol that was converted to geranyl acetate. Then, glucose supply was stopped at 48 h and the culture was continued under the glucose starvation condition. As expected, geranyl acetate was converted to geraniol after 56 h of culture while OD_600_ value of the bacterial culture began to decline slightly. At the end of fermentation, the concentration of geraniol reached maximum (2.0 g/L), and the yield (from glucose to geraniol) was 14 % which is approximately 11-fold that reported before [5]. Geranyl acetate production was reduced to 0.16 g/L at 68 h. Geraniol or neral was not detected while nerol was kept at a very low concentration during fermentation (0.05 g/L at 52 h). About 1.27 g/L (88.8 %) geranyl acetate was successfully converted to geraniol during the later stage of fermentation by glucose starvation.

Although higher concentration of geraniol was obtained in this study, geraniol production needs to be elevated at least 3 to 4 times in the future to meet the requirements of industrialization. Of all possible improvement strategies, engineering of the host can be realized by employing a chromosome integration technique to decrease the cell growth burden that results from overexpression of heterologous genes. Another approach is optimization of fermentation conditions such as induction time, inoculum age, and organic solvent for geraniol production.

## Conclusions

In summary, an efficient strain LWG6 was constructed and an isopropyl myristate-overlaid two-phase fed-batch fermentation system was established to biosynthesize geraniol from glucose. For the first time, the interconversion between geraniol and geranyl acetate by *E. coli* was observed, and Aes from *E. coli* was involved in the hydrolysis of geranyl acetate. Geraniol production reached up to 2.0 g/L with biotransformation of 88.8 % geranyl acetate to geraniol under controlled fermentation condition, which is the highest from glucose hitherto. This study provided a new strategy for geraniol biosynthesis.

## Methods

### Medium and culture conditions

Luria broth (LB) medium (10 g/L tryptone, 10 g/L NaCl, and 5 g/L yeast extract) was used for gene cloning and shake-flask fermentation. For geraniol production, recombinant strains were cultured in shake-flask or fed-batch fermentation with the medium containing 20 g/L glucose, 9.8 g/L K_2_HPO_4_, 5 g/L beef extract, 0.3 g/L ferric ammonium citrate, 2.1 g/L citric acid monohydrate, and 0.06 g/L MgSO_4_ as well as 1 ml of trace element solution which included 0.37 g/L (NH_4_)_6_Mo_7_O_24_·4H_2_O, 0.29 g/L ZnSO_4_·7H_2_O, 2.47 g/L H_3_BO_4_, 0.25 g/L CuSO_4_·5H_2_O, and 1.58 g/L MnCl_2_·4H_2_O. Appropriate antibiotics were added to the culture medium according to selectable marker gene of each plasmid listed in Table 1 at the following concentrations: ampicillin (Amp, 100 mg/ml), kanamycin (Kan, 50 mg/ml), and chloramphenicol (Cm, 34 mg/ml).

**Table 1:** Strains and plasmids used in this study

Name	Relevant characteristics	References
primers^a^		
GES-rbs-F	GGAAGATCTAGGAGGTAAAAAATATGTCTTGCGCTCGTATCACCG	This study
GES-R	CCGCTCGAGTTACTGGGTGAAGAACAGAGCG	This study
Aes-F-*Nco*I	CCCATGGCTATGAAGCCGGAAAACAAACT	This study
Aes-R-*EcoR*I	GGAATTCCTAAAGCTGAGCGGTAAAGAACTG	This study
Strains		
BL21(DE3)	F^−^ *omp* T, *hsd*S_B_ (r_B_^−^m_B_^−^), *gal*, *dcm me*131, λ(DE3)	Invitrogen
LWG6	*E.coli* BL21(DE3)/pLWG6, pYJM14	This study
LWG10	*E.coli* BL21(DE3)/p YJM26, pYJM14	This study
LWG11	*E.coli* BL21(DE3)/p LWG11	This study
Plasmids		
pET-30a	F1 (pBR322), Kan	Novagen
pGH/GES	pGH carrying GES from *O. basilicum*	This study
pLWG 6	pACYCDuet-1 carrying *mvaE* and *mvaS* from *E. faecalis*, *GPPS2* from *A. grandis*,GES from *O. basilicum*, Cm	This study
pLWG11	pET-30a carrying *aes* from *E. coli*, Kan	This study
pYJM26	pACYCDuet-1 carrying *mvaE* and *mvaS* from *E. faecalis*, *GPPS2* from *A. grandis*, Cm	[27]
pYJM14	pTrcHis2B carrying *ERG12*, *ERG8*, *ERG19* and *IDI1* from *S.cerevisiae*, Amp	[27]

^a^ Restriction sites are underlined

### Strains and plasmids

All strains and plasmids used in this study are listed in Table 1. The nucleotide sequences of *GES* gene from *O. basilicum* (sweet basil) (GenBank No. AY362553.1) were analyzed (http://www.genscript.com/cgi-bin/tools/rare_codon_analysis) and optimized to the preferred codon of *E. coli* (http://www.jcat.de/) online. The codon-optimized *GES* gene was synthesized by Genray Company with plasmid pGH as vector pGH/GES. *GES* gene was PCR-amplified from plasmid DNA of pGH/GES with primer set GES-rbs-F/GES-R. The PCR products were digested with *Bgl*II/*Xho*I, respectively, and introduced into the corresponding sites of pYJM26 to create pLWG 6. Plasmids pLWG 6 and pYJM14 were co-expressed in *E. coli* BL21 (DE3) to form strain LWG6. Aes was PCR-amplified from genomic DNA of BL21 (DE3) with primer set Aes-F-*Nco*I/Aes-R-*EcoR*I. The PCR product digested with *Nco*I and *EcoR*I was cloned into pET30a cut with the same restriction enzymes, creating pLWG11.

### Enzyme extraction and assay

LWG11 was cultured in LB broth and induced by IPTG at a final concentration of 0.1 mM when OD_600_ of the bacterial culture reached 0.6–0.8. After being incubated at 30 °C for 4–6 h, the cells were harvested by centrifugation at 6000 *g* for 5 min, washed with distilled water, and then resuspended in 0.5 M Tris-HCl (pH 8). All the extraction procedures were carried out at 4 °C. The cells were broken by sonic treatment for 10 min at 0 °C (3 s pulse on, 3 s pulse off, 40 W, Sonics VCX130, China). The Aes activity was determined by GC-MS monitoring of geraniol produced by the hydrolysis of geranyl acetate. The assay system consisted of 0.05 M Tris-Cl, pH 8.0, 5 mM MgSO_4_, 1 mM DTE, 2 mM geranyl acetate, and the enzyme extract (about 0.4 mg protein) in a total volume of 0.5 ml. The reaction mixture was incubated at 30 °C in a sealed capped tube for 2 h. A blank control with boiled enzyme was also run simultaneously.

### Shake-flask cultures

A single colony of LWG6 was grown in LB broth overnight at 37 °C. The culture was used to inoculate the same medium (1:100 dilution) and grown at 37 °C until an OD_600_ of 0.6–0.8 was reached. IPTG was added to a final concentration of 0.1 mM, and the culture was further incubated at 30 °C for 48 h. The samples were added with the same volume of ethyl acetate, vortexed briefly, and centrifuged to separate the phases, and the organic phase was analyzed by GC-MS. LWG10 strain was used as control. The experiment was performed in triplicate.

### Fed-batch fermentation for geraniol biosynthesis from glucose

LWG6 strain was grown overnight at 37 °C in 100 ml of LB medium and inoculated to a 5-L fermentor (BIOSTAT B plus MO5L, Sartorius, Germany) containing 2 L of fermentation medium. The temperature was maintained at 37 °C, and pH was maintained at 7.0 by automatically adding ammonia. Antifoam 204 was used to prohibit foam development. The stirring speed was first set at 400 rpm to maintain a 20 % saturation of dissolved oxygen. The expression of plasmid-born exogenous gene(s) for geraniol production was induced with 0.5 mM IPTG at 30 °C. During the course of fermentation, residual glucose was measured using a glucose analyzer (SBA-40D, China) and maintained below 5 g/L by a feeding solution containing 800 g/L glucose at appropriate rates. The samples were added with the same volume of ethyl acetate, vortexed briefly, and centrifuged to separate the phases, and the organic phase was analyzed.

To prevent violation, isopropyl myristate was added 4 h after IPTG induction according to one over ten of the fermentation medium volume. Glucose was stopped feeding after 48 h of culture. The samples were collected on time, and the organic phase was separated by centrifugation at 13,000 rpm for 10 min, then added with ten volumes of ethyl acetate and analyzed by GC-MS.

### Geranyl acetate feeding experiments

About 300 mg per liter medium of geraniol was fed to the cultures of *E. coli* BL21 (DE3) or LWG10 (OD_600_ at 20) to investigate the fate of geraniol during fermentation. Other fermentation conditions were the same as above.

### Geraniol characterization by GC-MS

Putative geraniol products were identified by GC-MS. A HP-INNOWAX capillary column (30 m × 0.25 mm; 0.25-μm-film thickness; Agilent Technologies) was used. The oven temperature was initially held at 80 °C for 1 min and sequentially increased at the rate of 10 °C/min to 180 and 30 °C/min to 250 °C. Peak identification was based on a relative retention time and total ion mass spectral comparison with an external standard (Sigma-Aldrich, USA). The peak areas were converted into concentrations in comparison with standard curves plotted with a set of known concentrations of standards.

## Abbreviations

Aes: acetylesterase
MVA: mevalonate
GPP: geranyl diphosphate
GPPS: geranyl diphosphate synthase
FPPS: farnesyl diphosphate synthase
GES: *Ocimum basilicum* geraniol synthase
Amp: ampicillin
Kan: kanamycin
Cm: chloramphenicol
IPTG: isopropyl *β*-d-thiogalactoside
