# Erratum to: Engineering *Escherichia coli* for high-yield geraniol production with biotransformation of geranyl acetate to geraniol under fed-batch culture

**DOI:** 10.1186/s13068-016-0539-5

**Published:** 2016-06-27

**Authors:** Wei Liu, Xin Xu, Rubing Zhang, Tao Cheng, Yujin Cao, Xiaoxiao Li, Jiantao Guo, Huizhou Liu, Mo Xian

**Affiliations:** CAS Key Laboratory of Bio-Based Materials, Qingdao Institute of Bioenergy and Bioprocess Technology, Chinese Academy of Sciences, Qingdao, 266101 China; Key Laboratory of Green Process and Engineering, Institute of Process Engineering, Chinese Academy of Sciences, Beijing, 10090 China; University of Chinese Academy of Sciences, Beijing, 100049 China; Department of Chemistry, University of Nebraska-Lincoln, Lincoln, NE 68588 USA

## Erratum to: Biotechnol Biofuels (2016) 9:58 DOI 10.1186/s13068-016-0466-5

Following publication of the original article in *Biotechnology for Biofuels* [1], it was brought to our attention that the following sentences are incorrect:

“At the end of fermentation, the concentration of geraniol reached maximum (2.0 g/L), and the yield (from glucose to geraniol) was 14 % which is approximately 11-fold that reported before [5].”

This sentence should read: At the end of fermentation, the concentration of geraniol reached maximum (2.0 g/L), and the yield (from glucose to geraniol) was 14 % of the theoretical yield, which is approximately 11-fold that reported before [5].

“Geraniol or neral was not detected while nerol was kept at a very low concentration during fermentation (0.05 g/L at 52 h).”

This sentence should read: Geranial or neral was not detected while nerol was kept at a very low concentration during fermentation (0.05 g/L at 52 h).

The header “Geranyl acetate feeding experiments” should read: Geraniol feeding experiments.

Furthermore, the captions of Figs. 3 and 4 are incorrect.

The caption of Fig. 3 “Geranyl acetate feeding experiments by *E. coli* BL21 (DE3)”, should read: Geraniol feeding experiments by *E. coli* BL21 (DE3).

The caption of Fig. 4 “Geranyl acetate feeding experiments by LWG10”, should read: Geraniol feeding experiments by LWG10.

We apologize for the inconvenience this may have caused.
